# Metric and Spectral Insight into Bee-Pollen-to-Bee-Bread Transformation Process

**DOI:** 10.3390/foods12224149

**Published:** 2023-11-16

**Authors:** Lidija Svečnjak, Kristian Bošković, Saša Prđun, Mirna Mrkonjić Fuka, Irina Tanuwidjaja

**Affiliations:** 1 Department of Fisheries, Apiculture, Wildlife Management and Special Zoology, Faculty of Agriculture, University of Zagreb, Svetošimunska Cesta 25, 10000 Zagreb, Croatiasprdjun@agr.hr (S.P.); 2 Department of Microbiology, Faculty of Agriculture, University of Zagreb, Svetošimunska Cesta 25, 10000 Zagreb, Croatia; mfuka@agr.hr (M.M.F.); irinawijaya@gmail.com (I.T.); 3 Institute of Sanitary Engineering and Water Pollution Control, Department of Water, Atmosphere and Environment, University of Natural Resources and Life Sciences, Muthgasse 18, 1190 Vienna, Austria

**Keywords:** bee pollen, bee bread, bee-pollen-to-bee-bread transformation process, honeycomb cell depth, FTIR-ATR spectral profiles

## Abstract

Due to numerous bioactive constituents, both bee pollen (BP) and bee bread (BB) represent valuable food supplements. The transformation of BP into BB is a complex biochemical in-hive process that enables the preservation of the pollen’s nutritional value. The aim of this study was to determine the depth of the honeycomb cells in which bees store pollen and to provide a spectral insight into the chemical changes that occur during the BP-to-BB transformation process. This study was carried out on three experimental colonies of *Apis mellifera* carnica, from which fresh BP was collected using pollen traps, while BB samples were manually extracted from the cells two weeks after BP sampling. The samples were analyzed using infrared (FTIR-ATR) spectroscopy, and the depth of the cells was measured using a caliper. The results showed that the average depth of the cells was 11.0 mm, and that the bees stored BB up to an average of 7.85 mm, thus covering between ⅔ and ¾ (71.4%) of the cell. The FTIR-ATR analysis revealed unique spectral profiles of both BP and BB, indicating compositional changes primarily reflected in a higher water content and an altered composition of the carbohydrate fraction (and, to a lesser extent, the lipid fraction) in BB compared to BP.

## 1. Introduction

Due to numerous bioactive constituents, both bee pollen and bee bread represent a rich source of nutrients and could be beneficial food supplements. Moreover, nowadays, they stand out as an emblematic example of underutilized sources of bioactive compounds [1]. Of all bee products, bee bread is the least researched and utilized, although it has been proven to exhibit health-promoting properties, such as antimicrobial, antioxidant, antiradical, and anti-inflammatory activities [2,3,4,5,6,7,8,9,10,11,12].

The production of bee pollen, and consequently bee bread, is a complex biological process that represents and starts with a unique mode of synergy of plants and honey bees (*Apis mellifera* L.). When performing their pollination services for numerous plant species, honey bees collect pollen from the anthers of the plants on their hairs, mix it with a small amount of their salivary gland secretions and nectar, place it into specialized organs (pollen basket) on their hind legs, and carry it to the hive where the bee pollen is extruded into the honeycomb cells, where it is further processed (subjected to fermentation) for conservation purpose. Such bee-stored pollen serves as the main source of proteins, lipids, minerals, and vitamins in the honey bees’ nutrition [13]. Prior to in-hive storage, the pollen grains collected by the bees are agglutinated in the pollen basket by moistening with bee salivary secretions and nectar, which results in the formation of a pellet made of pollen grains, whose size ranges from 1.4 to 4.0 mm [14,15]. This pellet form of the field-foraged pollen placed in the pollen baskets is known as bee pollen or pollen load.

The chemical composition of pollen varies with the plant species, the environment during pollen development, and the age of plant when the pollen developed, as well the nutrient status of the plant [13], which makes bee pollen equally variable in composition [14,16,17,18]. Also, this large variability in the chemical composition of pollen results in an equally large variability in its nutritional value for the honey bees [13]. The chemical compositions of bee pollen have drawn intensive worldwide research attention, especially in the last two decades. As overviewed by Thakur and Nanda [14], based on a systematic review of 100 studies, bee pollen contains on average 54.22% (18.50–84.25%) carbohydrates, 21.30% (4.50–40.70%) proteins, 5.31% (0.41–13.50%) lipids, and 8.75% (0.15–31.26%) fiber, while other bee pollen constituents are represented in smaller, but still considerable, amounts, such as phenolic compounds, vitamins, minerals, volatile compounds, and enzymes [16,18,19,20,21,22]. It has been generally stated that the moisture content in bee pollen varies from 1.5 to 13% [1], although fresh bee pollen contains on average 17.5% moisture, and often more than 20% moisture [14,16].

Bee pollen is a well-balanced food for the honey bee colony, however, its composition makes it susceptible to rapid degradation under the in-hive conditions (35 °C). Therefore, honey bees have developed a mechanism to stop the in-hive deterioration of bee pollen and preserve its nutritional value by converting it into so-called bee bread. The transformation of bee pollen into bee bread represents a natural biochemical in-hive process that takes place directly in the honeycomb cells. Namely, the worker bees cover the bee pollen stored in the cells with a thin layer of honey and add salivary gland secretions containing various enzymes [3,23,24].

Altogether, this triggers a microbial transformation of BP to BB, which is carried out by three distinctive groups of microbes. First, aerobic bacteria, such as *Pseudomonas* spp., predigest the bee pollen and consume oxygen, thus creating anaerobic conditions. Lactic acid bacteria (LAB), primarily *Streptococcus* spp. and *Lactobacillus* spp., takeover and ferment the carbohydrates into lactic acid, increasing the acidity and creating favorable conditions for the growth of microscopic fungi. Finally, *Saccharomyces* spp. metabolize the remaining carbohydrates [25]. Although the exact mechanism of the biotransformation of BP to BB is not yet fully understood, LAB are mainly involved in the fermentation and stabilization of BB, while *Pseudomonas* spp. and *Saccharomyces* spp. might play a central role in the degradation of pollen walls and membranes [26]. After 15 days of transformation, the microbial diversity in the BB is greatly reduced compared to that of the pollen, and the pH of BB is around 4, which allows the conservation of BB by slowing the growth of spoilage microorganisms [3,5,25].

The lower pH value of bee bread, caused by the lactic acid fermentation, in addition to the presence of various digestive enzymes (such as invertase, aminopeptidase, phosphatase, β-glucosidase, amylase, phosphatase, glucose-oxidase, and others) [3,5,27] contributes to the higher nutrient bioavailability of bee bread compared to bee pollen [28,29], as well as better beneficial/protective effects in terms of antimicrobial activity [2,4,6,9,10,30]. Compared to bee pollen, the chemical composition of bee bread has been explored to a lesser extent, therefore, this bee product can still be considered as insufficiently investigated. Given that the chemical composition of bee bread is the result of the fermentation of bee pollen, its composition differs compared to that of bee pollen, which is primarily manifested in a lower proportion of carbohydrates and a higher proportion of water, proteins, and ash [1].

As elaborated above, honeycomb represents a natural ripening media in which bee pollen is converted into more stable and more beneficial/bioavailable bee bread. Based on old, but still valid, literature data, Bader et al. [31] stated that *A. mellifera* honeycomb is typically constructed from a series of hexagonal cells with edge lengths ranging from ca. 2.25 to 2.75 mm, an edge thicknesses of ca. 0.75 mm, and average depths ranging from ca. 10 to 11 mm. The mode of bee pollen storage in the honeycomb cells is generally known; however, this is based on a visual approximation. It has been mostly stated that honey bees store bee pollen in up to 34 of the cell [7,32], or that the bee pollen stored in the cells occupies about 23 of the cell [33]. However, to the best of our knowledge, there are no exact metric data on the depth of the honeycomb cells in which honey bees store their collected pollen loads (data on the bee bread storage depth) in the available scientific literature. In addition, the background mechanisms of the chemical conversion of bee pollen into bee bread based on the simultaneous investigation of both substances have not yet been investigated sufficiently. Therefore, the aim of this study was to determine the depth of the honeycomb cells in which honey bees store pollen during the bee-pollen-to-bee-bread transformation process and to provide a spectral insight into the chemical changes in the pollen composition from the initial raw material (bee pollen) to the final product (bee bread).

## 2. Materials and Methods

### 2.1. Experimental Design

This study was carried out on three experimental colonies of Carniolan honey bees (*Apis mellifera carnica*, Pollmann, 1879) placed in Langstroth-Rooth hives (H1, H2, and H3) and situated at the experimental apiary of the University of Zagreb Faculty of Agriculture. The experimental colonies were uniformed by strength according to the method described by Delaplane et al. [34] prior to the experiment, which was conducted during the April–May period of the production season in 2021. Front-mounted pollen traps were placed at the entrance of the experimental hives to ensure the collection of bee pollen samples. In addition, frames with honeycombs containing empty worker cells were placed in each experimental colony (in a honey super at lateral positions) to enable monitoring and storage of the pollen load in the cells, as well as further sampling and analysis of both the honeycomb and bee bread.

### 2.2. Sampling of Bee Pollen and Bee Bread

The bee pollen samples (BP; *n* = 3; 1 pooled sample/experimental colony) were collected using front-mounted pollen traps placed at the entrance of the experimental hives. A frame with stored bee pollen was pooled out from each experimental honey bee colony two weeks after bee pollen sampling, and individual bee bread samples were manually extracted directly from the honeycomb cells (BB; *n* = 105; 35 samples/experimental colony) using a small spatula. The samples of BP were stored in small glass containers (25 mL) and the BB samples were stored in Eppendorf tubes (1 mL) at −18 °C until analysis.

### 2.3. Determination of the Cell Depth of Bee Bread Storage and Complementary Cell Measurements

**Figure 1:**
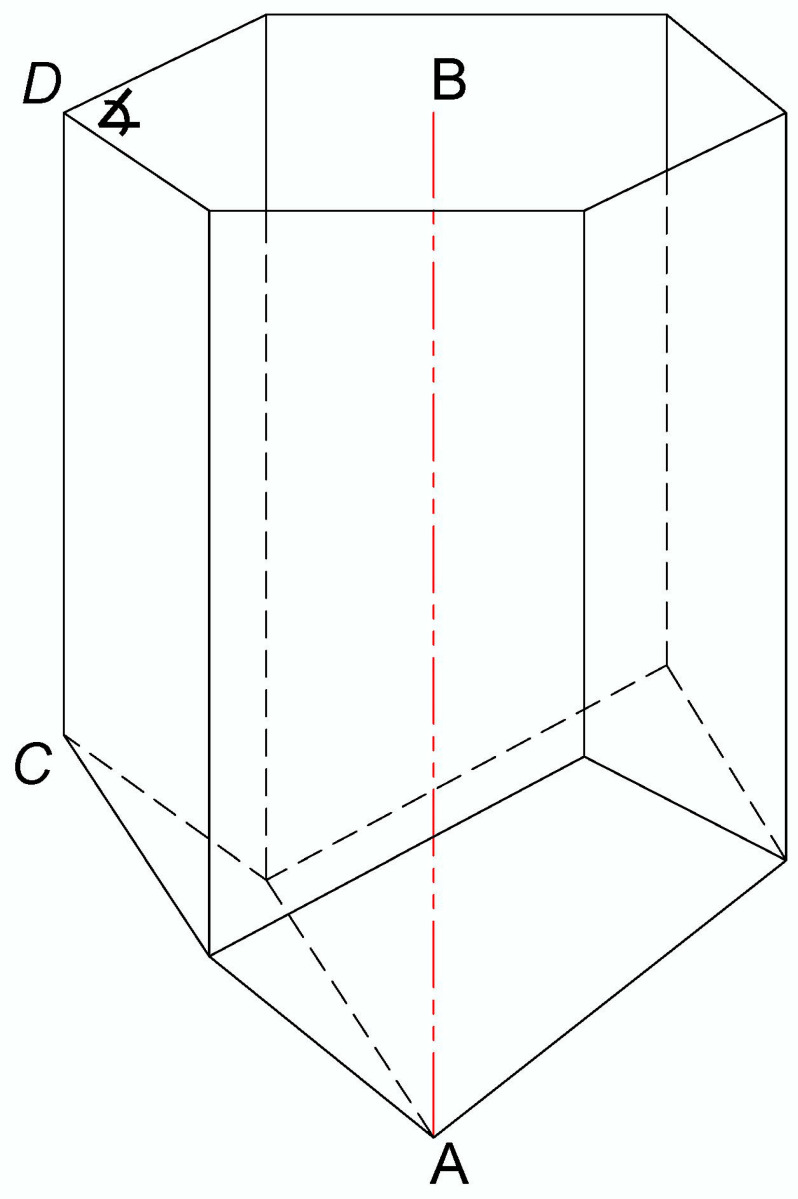
A three-dimensional view of the honeycomb cell representing conducted cell depth measurement along the A–B length and complementary measurements of the smallest side length (*C*–*D*) and the angle (∡) of the hexagonal cell.

The frames with honeycombs that were isolated from each experimental colony and used for BB sampling were also used for obtaining the metric data on the pollen storage, i.e., for the determination of the depth of the honeycomb cell in which the honey bees store bee bread. The honeycombs were cut with a scalpel to obtain a fine cross-section, which enabled precise measurements. The total depth of the honeycomb cells (*n* = 30/colony) and the depth of the cells in which the bee bread was stored (*n* = 100/colony) were measured in accordance with Svečnjak et al. [35] methodology, whereas the method of measuring the longest possible length along A–B (deepest point in the cell) was chosen, as presented in Figure 1. In addition to measuring targeted cells filled with BB, the total depth of the cells was also measured as an auxiliary measure for determining the average depth of *A. m. carnica* bee bread storage in relation to the total cell depth. Additional complementary measurements of the honeycomb cells, i.e., measurement of the smallest side length along *C*–*D* and the angle of the hexagonal cell (*n* = 30/colony), were also performed to obtain additional data on the geometrical properties of *A. m. carnica* honeycomb cells in which BP and BB are stored.

The measurements of the depth and side length of the honeycomb cells were carried out using a caliper (Traceable Digital Caliper, Fisherbrand^TM^, UK, measuring range: 0–150 mm, resolution: 0.01 mm), while the angle of the hexagonal honeycomb cells was measured using a precise digital protractor (Leman, Saint-Clair-de-la-Tour, France, measuring range: 0°–360°, resolution: 0.05°).

### 2.4. Statistical Analysis

In order to assess the differences in the cell depth between the three experimental colonies, as well as the distribution of bee bread according to the depth of its storage in the honeycomb cells, data were analyzed by means of descriptive statistics, classical one-way analysis of variance (ANOVA), and Kruskal–Wallis one-way ANOVA. The mentioned data analyses were performed using the statistical software package Statistica-StatSoft v.7. [36].

### 2.5. FTIR-ATR Spectroscopy and Spectral Data Analysis

The collected BP and BB samples were analyzed using Fourier transform infrared spectroscopy (FTIR) coupled with an attenuated total reflectance (ATR) accessory. For this purpose, a Cary 660 FTIR spectrometer (Agilent Technologies, Palo Alto, CA, USA) coupled with a Golden Gate single-reflection diamond ATR accessory (Specac) was used. Prior to the spectral analysis, the BP samples were pulverized with a porcelain mortar into homogenates, while the BB samples were analyzed as obtained. To obtain spectra, approximately 3 mg of sample was pressed onto a diamond ATR plate using a self-leveling sapphire anvil in order to ensure a thin uniform layer of each sample. The absorption infrared (IR) spectra of the BP and BB samples were recorded in a mid-infrared region (4000–400 cm^−1^). Spectra were acquired at a nominal resolution of 4 cm^−1^ and at room temperature (24 ± 2 °C). Two replicate spectra (32 scans/spectrum) of each BP and BB sample were recorded using different aliquots. The spectra were recorded and pre-processed using the Resolutions Pro version 5.3.0 software package [37], while further spectral data analysis was carried out using Origin version 8.1 [38]. Qualitative analysis and the identification of analyte signals observed in the BP and BB spectra were performed using spectral atlases and the available scientific literature. In order to elucidate overlapping spectral effects related to water signals, a complementary analysis was performed on the BP and BB samples that were lyophilized (as described below) and reanalyzed with the same FTIR-ATR procedure. Also, for the same purpose, water loss was determined gravimetrically.

### 2.6. Freeze-Drying (Lyophilisation) of BP and BB Samples

Freeze-drying of the BP and BB samples was carried out at −50 °C (under 1 mbar pressure) over 24 h by using an Alpha 1-4 LSCplus laboratory freeze-dryer (Christ, Osterode am Harz, Germany). In addition, water loss was determined gravimetrically. For this purpose, 2 g of BP and BB (pooled) samples from H1, H2, and H3 were weighed before lyophilization and reweighed after this treatment by using a precise analytical balance MS 204 Mettler Toledo (Columbus, OH, USA).

## 3. Results and Discussion

### 3.1. Determination of the Cell Depth of Bee Bread Storage

In order to estimate the depth of the bee bread storage in relation to the total depth of the honeycomb cells, the latter was also determined. As presented in Table 1, the average total depth of the honeycomb cells determined in experimental colony H1 was 10.99 mm, 11.01 mm in H2, and 11.01 mm colony H3, which reflects a great similarity of average values and confirms the well-known uniform architecture of honeycomb cells. The determined maximum values of the cell depth in the three experimental honey bee colonies also revealed high resemblance, at 12.05 mm, 11.98 mm. and 12.36 mm, respectively, while the most noticeable differences between the colonies (although not statistically significant, as noted below) were observed for the minimum cell depth values (9.71 mm determined in the H1 colony, 8.97 mm in the H2 colony, and 9.37 mm in H3 colony).

**Figure 2:**
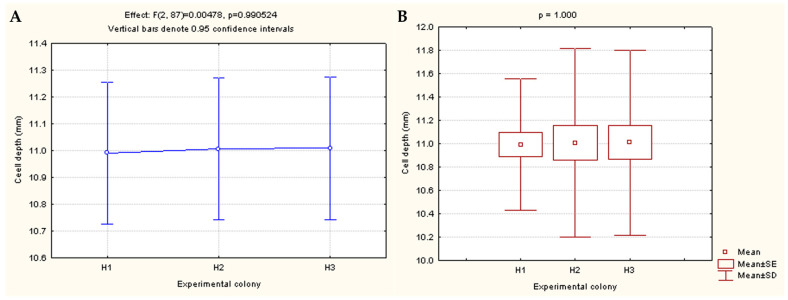
Effects of one-way ANOVA showing differences in the depth of *A. m. carnica* honeycomb cells between three experimental colonies (H1, H2, and H3) (**A**); Differences presented via effects/box and whisker plot of Kruskal–Wallis one-way ANOVA (**B**).

As presented in Figure 2, the results of the one-way ANOVA showed no statistically significant differences between the colonies with regard to the measured depth of the honeycomb cells (*p* = 0.990524).

The overall results showed that the average depth (mean ± SD) of *A. m. carnica* honeycomb cells was 11.00 ± 0.72 mm. This is in compliance with the old literature data reporting 11.00 mm as an average cell depth of *A. mellifera* worker cells [39], as well as the recent data on the cell depth (ranging from 9.23 to 13.06 mm, depending on the comb age, on average 11.11 mm) reported for honeycomb constructed by *A. m. carnica* [40]. The obtained results are also similar to those reported for the average depth of the standard brood cells of European dark bees (*Apis mellifera mellifera*), i.e., 11.03 ± 0.64 [41]. Compared to the available scientific literature reporting on the cell dimensions of other bee species, moderate differences were observed. As reported by Yang et al. [42], the average depth of the worker cells determined for Chinese bees (*Apis cerana cerana*) was 9.595 mm ± 0.066 mm and was 12.088 mm ± 0.040 mm for Italian bees (*Apis mellifera ligustica*). The data on the cell depth reported for Africanized honey bees (*Apis mellifera scutelata*) investigated in two agro-ecological systems in Western Ethiopia revealed that the cell depth in the highland and mid-altitude region ranged from 9.6 mm to 11.4 mm, respectively [43].

As shown in Table 2, the average depth of the honeycomb in which the honey bees stored bee bread ranged from 7.38 mm in the H1 colony to 8.02 mm in H3 and 8.14 mm in experimental colony H2. The minimum depth of the bee bread storage was 5.19 mm in colony H1, 5.25 mm in colony H2, and 5.20 mm in colony H3, while the maximum depth of the comb cells in the H1 colony was 10.26 mm, 10.71 mm in H2, and 9.72 mm in the H3 colony.

**Figure 3:**
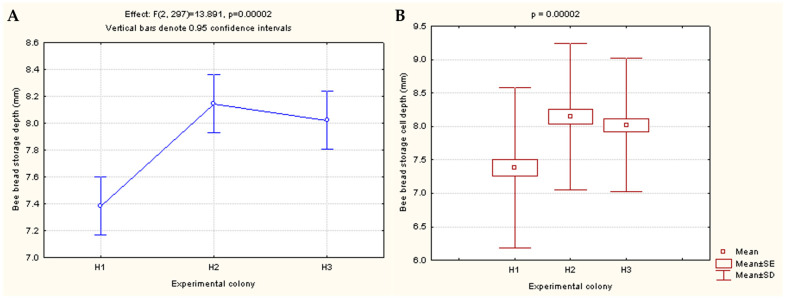
Effects of one-way ANOVA showing differences in the cell depth of bee bread storage between three experimental colonies (H1, H2, and H3) (**A**); Differences presented via effects/box and whisker plot of Kruskal–Wallis one-way ANOVA (**B**).

The results of the one-way ANOVA showed statistically significant differences between the colonies with regard to the bee bread storage depth (*p* = 0.00002) (Figure 3).

**Figure 4:**
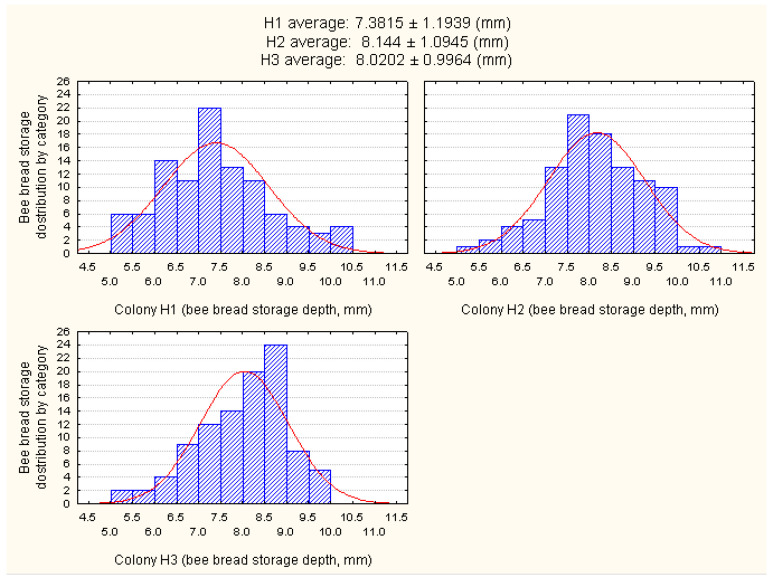
Categorized histogram showing a distribution of bee bread according to the depth of its storage in the honeycomb cells of three experimental colonies (H1, H2, and H3).

Differences in the mode of bee bread storage were also observed. A distribution of bee bread according to the depth of its storage in the honeycomb cells of the three experimental colonies (H1, H2, and H3) is presented in Figure 4.

The results have revealed that most of the bee bread was stored between 6.5 and 9.5 mm of the cell, with an average of 7.85 mm. Part of the results related to the lower values of bee bread storage (<6.5 mm) observed in all three of the experimental colonies can be explained by the simultaneous consumption of bee bread by the honey bees during the experiment, as they tend to consume freshly stored pollen as well [44].

To the best of our knowledge, there are no exact metric data about the depth of bee bread storage in the available scientific literature. This may be explained by the fact that the measurements of the depth and/or estimations of the internal volume of the cell represent a demanding task due to the unique hexagonal cell geometry. The mode of bee pollen storage in the honeycomb cells is generally known, and it has been mostly stated that honey bees store bee pollen in up to 34 of the cell [7,32], or that the bee pollen stored in the cells occupies about 23 of the cell [33]. Thus, the information about the cell depth in which bee pollen/bee bread is stored in the honeycomb cells has been simplified/generalized based on a visual approximation, which implies that bee bread occupies between 66.6% (23) and 75.0% (34) of the cell.

**Figure 5:**
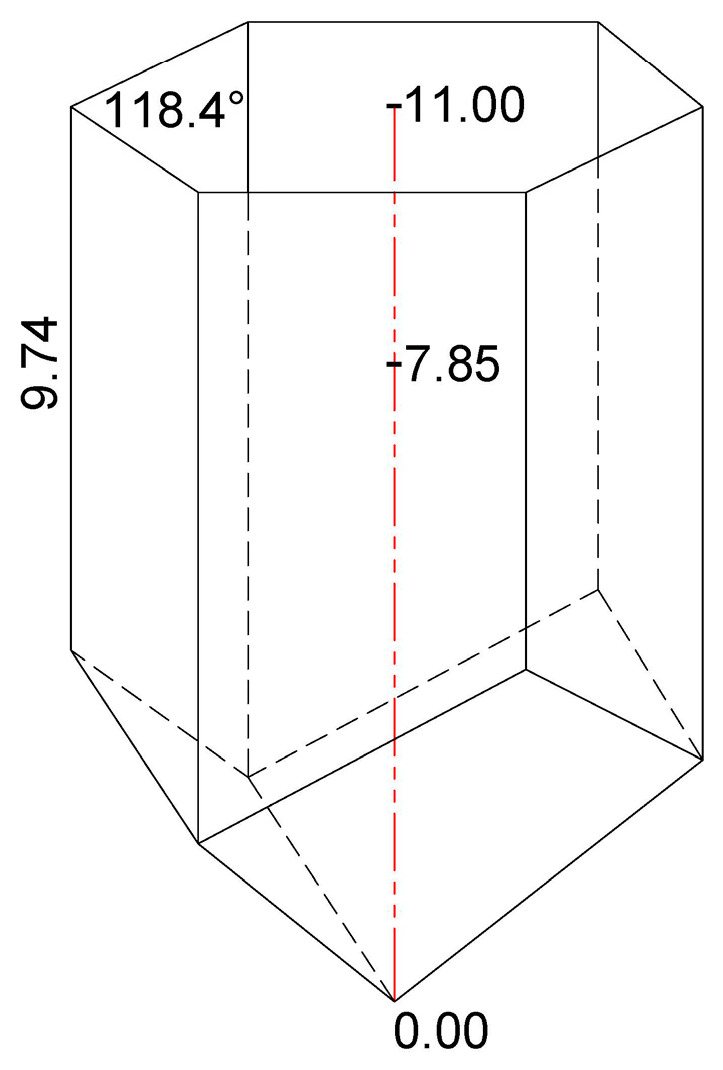
A three-dimensional view of the honeycomb cell representing the obtained results on the total cell depth (11.00 mm) and the depth of bee bread storage (7.85 mm) in *A. m. carnica* honeycomb, along with results on complementary measurements (average values of the smallest side length and the angle of the hexagonal cell).

The overall metric data obtained in this study have revealed that the average depth of the honeycomb cells constructed by the *A. m. carnica* workers was 11 mm, and that the bees stored bee bread up to an average of 7.85 mm of cell depth (Figure 5), thus covering between 23 and 34 (71.4%) of the honeycomb cell.

To be more specific, in terms of the usual representation of bee bread storage in the form of arithmetic fractions in relation to the total cell depth, the closest value implied by the results of this study would be 1724 of the cell depth. To the best of our knowledge, this finding represents the first report, i.e., the first metric data, on the cell depth in which *A. m. carnica* honey bees store bee bread. These data may be employed to investigate the hive-level distribution and storage of bee bread and to estimate the annual production and economic value of bee bread production in general. 

Figure 5 also shows the results of the complementary measurements of the smallest side length and the angle of the hexagonal cells obtained for the analyzed honeycombs (average values based on *n* = 30 cells/experimental colony H1, H2, and H3). The results have revealed that the smallest side length of the analyzed honeycomb cells ranged from 9.12 to 10.63 mm, with an average of 9.74 mm, while the angle of the hexagonal cells ranged from 117.8° to 119.2°, with an average of 118.4° (Figure 1). The results have revealed a great similarity of the smallest side length to the angle of the hexagonal cells, both within and between the colonies, thus reflecting consistency and uniformity in *A. m. carnica* honeycomb construction. These complementary metric data represent an additional insight into the geometrical properties of *A. m. carnica* honeycomb cells where the BP-to-BB transformation pathway occurs and may also be used for a further investigation into BP/BB storage in the honeycomb cells, as well as other cell-related studies. The descriptive statistics for the smallest side length and the angle of the hexagonal cells for colonies H1, H2, and H3 are presented in Table 3 and Table 4, respectively.

### 3.2. Spectral Analysis of Bee Pollen and Bee Bread

FTIR-ATR analysis has been employed for BP or BB analysis in few recent studies. The first FTIR-ATR study of bee pollen was conducted by Anjos et al. [45], who reported on the usefulness of this analytical tool for studying the overall chemical composition of bee pollen. Further investigations on bee pollen composition using FTIR-ATR spectroscopy were conducted by several authors [16,17,46,47], among which Prđun et al. [16] and Castiglioni et al. [17] presented the most comprehensive spectral analysis of unifloral and/or multifloral bee pollen, as well as their physicochemical characterization. The only study dealing with FTIR analysis of bee bread was reported by Dranca et al. [48]. However, the assignment of vibrational modes presented in this study is actually based on the spectrum of bee pollen from another (previous) study of the same authors, and not on the spectrum of bee bread investigated in a respective study (i.e., spectrum of crude BP was presented and tentatively interpreted instead of BB spectrum, and the authors justified this by the ‘spectra resemblance’ of BP and BB). Such a presentation of the results of the spectral analysis of BB (based on FTIR analysis of another substance/BP, without presenting the BB spectrum itself) is misleading and raises the question of its eligibility. Also, it should be noted that only the integral spectral profiles of the BP and BB samples show a general resemblance, but the spectral differences between BP and BB are noticeable (as well as expected, considering the background chemical alterations of the BP-to-BB transformation pathway), which is presented and elaborated in our study.

**Figure 6:**
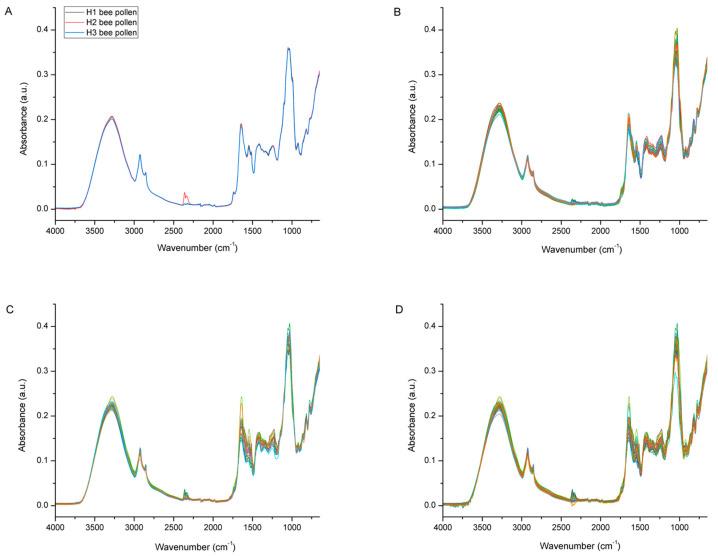
FTIR-ATR spectra of multifloral bee pollen (*n* = 1/colony) collected from experimental colonies H1, H2, and H3 (**A**); FTIR-ATR spectra of bee bread samples (*n* = 35/colony) collected from individual honeycomb cells of experimental colonies H1 (**B**), H2 (**C**), and H3 (**D**); different colors on (**B**–**D**) denote spectra of bee bread samples collected from individual honeycomb cells.

Figure 6 represents raw the FTIR-ATR spectra of multifloral bee pollen (*n* = 3; 1 pooled sample/colony) collected from the three experimental colonies (H1, H2, and H3) and the raw FTIR-ATR spectra of the bee bread samples (*n* = 35/colony) collected from individual honeycomb cells of the same experimental colonies. As presented in Figure 6A, the bee pollen collected from the different colonies showed a great similarity of spectral features, i.e., no noticeable differences in their chemical composition. This was expected, considering that the experimental colonies were situated in the same location during the same period (the honey bees will have collected available bee pollen of the same botanical origin, and, in addition, the samples were homogenized prior to the FTIR-ATR analysis). The general spectral profiles of the analyzed bee pollen correspond to those reported in previous studies of bee pollen using FTIR-ATR spectroscopy [16,17,45,46,47].

**Figure 7:**
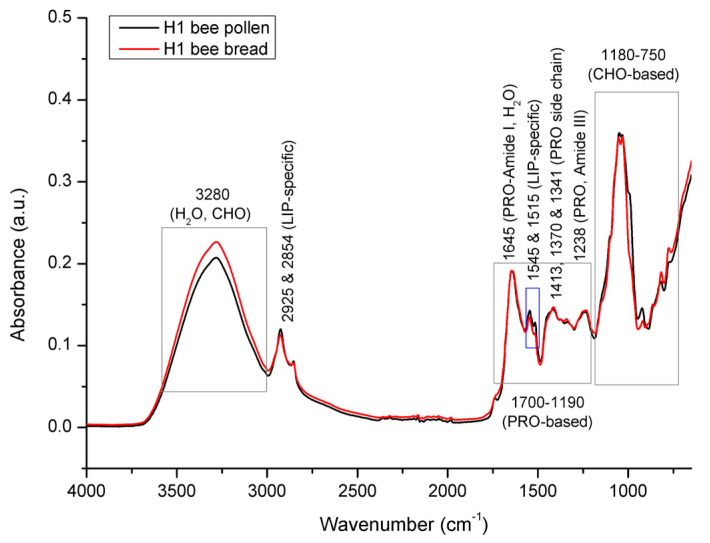
Average FTIR-ATR spectrum of bee pollen vs. bee bread from experimental colony H1, representing comparative spectral features with the assignation of major underlying molecular vibrations (whole spectral region from 4000 to 600 cm^−1^). CHO = carbohydrates; PRO = proteins; LIP = lipids.

**Figure 8:**
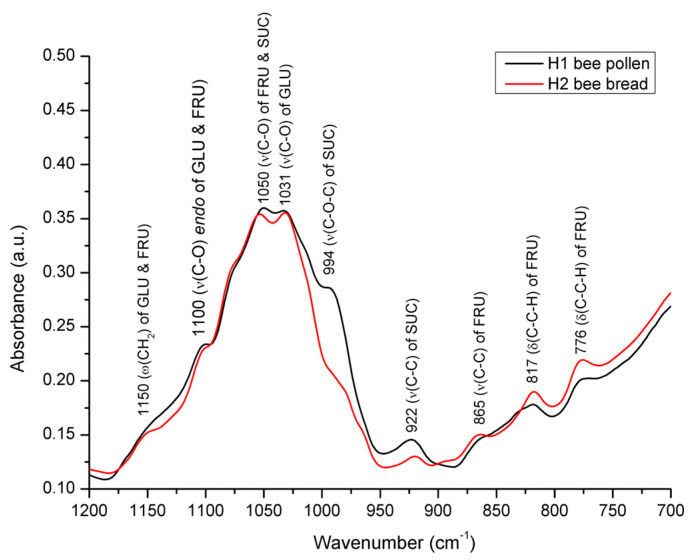
Average FTIR-ATR spectrum of bee pollen vs. bee bread from experimental colony H1 representing comparative spectral features with the assignation of major underlying molecular vibrations (carbohydrate-specific spectral region from 1200 to 700 cm^−1^). ν = *in-plane* stretching vibration; δ = *in-plane* bending vibration (deformation); ω = *out-of-plane* bending vibration (wagging); GLU = glucose; FRU = fructose; SUC = sucrose.

Figure 6B–D shows the FTIR-ATR spectra of the bee bread samples (*n* = 35/colony) collected from individual honeycomb cells from the experimental colonies H1, H2, and H3, respectively. Obvious spectral variations were observed throughout the entire spectral region of the bee bread samples collected from each colony, which indicates variability in the chemical composition of the individual bee bread samples. Those variations were most pronounced in the spectral region between 3600 and 3000 cm^−1^ (indicating alterations in the water and carbohydrate content), but were even more prominent in the fingerprint region between 1700 and 750 cm^−1^ (indicating variations related to the major BB constituents—sugars, proteins, and lipids). A detailed assignment of the individual bands observed in these regions is provided below in Figure 7 and Figure 8. The mentioned variations are the result of a specific way of storing the pollen load in the honeycomb cells, which causes inhomogeneity in the composition of the bee bread samples in individual honeycomb cells (pollen of various plant species is placed in the cells); however, part of such variations may also be explained by the different stage of fermentation of the bee bread in the individual cells [5].

The chemical characterization of both bee pollen and bee bread is provided further in Figure 7 and Figure 8 via a comparative overview of their spectral profiles, elucidating the chemical alterations that occur during the bee-pollen-to-bee-bread transformation pathway.

Figure 7 represents comparative spectral features of bee pollen (average spectra of pooled multifloral bee pollen sample/colony) and bee bread (average spectra based on *n* = 35 bee bread samples/colony) collected from experimental colony H1. Both bee pollen (BP) and bee bread (BB) represent a complex organic mixture of various constituents, including all major biological macromolecules (proteins, carbohydrates, and lipids). Consequently, their FTIR-ATR spectra exhibit a broad variety of absorption bands, as well as numerous overlapping spectral effects, arising from diverse molecular vibrations of these constituents. As summarized by Giampieri et al. [1], the composition of both BP and BB vary significantly. BP is composed of 1.5–13.8% water, 18.5–82.0% of carbohydrates, 2.5–62.0% of proteins, 0.41–24.40% of lipids, 0.15–30.0% of dietary fibers, and 0.5–6.5% of ash; while BB comprises 5.91–30.12% water, 13.02–72.23% of carbohydrates, 17.11–30.34% of proteins, 1.95–11.95% of lipids, and 1.93–3.42% of ash. Generally, there are a lot of data on the composition of bee pollen and bee bread individually in the available scientific literature, however, there is only one record on the simultaneous comparative analyses of both of these substances from the same hives [28]. However, in their study, Mayda et al. [28] concluded that the total protein, total fatty acids, and moisture content of the BB samples were lower than those of the BP samples from the same hive. This is not entirely in compliance with the results obtained in our study, as well as those from other reports investigating the total composition of BP and BB, as overviewed by Giampieri et al. [1]. To be more specific, our results (as presented in Figure 7), as well as those of other reports, indicate a higher content of moisture in BB compared to BP, while no noticeable differences in the proportion of proteins were found between BP and BB (which is expected, given that the major purpose of in-hive BP-to-BB conversion is the preservation of proteins and considering that BP is the main source of proteins for honey bees). The contradiction on the moisture content reported by Mayda et al. [28] can be explained by the rather vague and unfounded interpretation of the results on the moisture content obtained in the respective study, given that BP and BB were found to have similar moisture levels, i.e., the moisture content of BP was between 17.3% and 23%, while in BB it was between 17.7% and 22.3%. These data do not support the general conclusion that BB contains a lower moisture content, considering the presented range values, as well as the almost-equal average values (19.4% for BP and 19.06% for BB). In addition, the respective study was carried out on a small number of BP and BB samples (five BP and five BB pooled samples), which further calls into question the conclusion on the lower moisture content found in BB. It should also be emphasized that the organoleptic profile of BB vs. BP, as well as the fact that BP is moistened with a layer of honey during the BP-to-BB transformation process, implies a higher moisture content in BB compared to BP. The allegation on the proportion of lipids/fatty acids is in compliance with previous studies and our results.

As presented in Figure 7, the results of the spectral analysis have revealed that the most prominent spectral differences between BP and BB are manifested in the spectral range from 3600 to 3000 cm^−1^, and in a particular segment of the fingerprint region (1700–750 cm^−1^). The intensity of a broad and strong absorption band with an absorption maximum at 3280 cm^−1^ occurring in the spectral range from 3600 to 3000 cm^−1^ that is primarily assigned to O–H stretching vibrations of water and partially to carbohydrates [16,49,50,51], is higher in BB compared to that observed in BP, which primarily reflects a higher proportion of water in BB, and, partly, changes in the sugar fraction profile. It should be noted that an absorption band assigned to the N–H stretching vibrations of proteins (Amide A band) also typically arises in the same (3600–3000 cm^−1^) spectral region [51,52], but it is overlapped by the more intense vibrations of O–H groups of water and carbohydrates [16], which makes this region non-specific for the monitoring of proteins in the analyzed samples. 

Two IR analyte signals observed in the spectral region from 3000 to 2920 cm^−1^, the medium intensity signal arising at 2925 cm^−1^, and the weaker absorption band observed at 2854 cm^−1^ correspond to the stretching vibrations of the aliphatic C–H groups and/or CH_2_ groups, and can generally be related to the molecular vibrations of numerous bee pollen constituents, such as carbohydrates, cellulose, lipids, fatty acids, and other long-chain structures [16,17,45,46,47]. However, it can be concluded that the signals observed at 2925 cm^−1^ and 2854 cm^−1^ are primarily associated with the asymmetric and symmetric stretching vibrations of the CH_2_ groups of lipids (aliphatic chains), considering that triglycerides are the most abundant component of the lipid fraction in BP (followed by diglyceride and free fatty acids) [53], and they typically absorb IR radiation strongly in this region [35,51,54,55]. These signals were found to be stronger in BP compared to those in BB, which indicates a higher amount of lipids in BP compared to that of BB. This was confirmed by a stronger intensity of absorption bands arising at 1544 cm^−1^ and 1515 cm^−1^ that are also related to lipids, as explained below. It should be mentioned the C–H stretching vibrations of BP carbohydrates are also represented with two signals at positions similar to 2925 and 2854 cm^−1^ [16,45], but are overlapped by the mentioned molecular vibrations of lipids, as they exhibit more intensive signals compared to C–H stretching of carbohydrates in this spectral region.

As emphasized in Figure 7, the integral spectral profiles of BP and BB are very similar in most of the spectral regions between 1700 and 1190 cm^−1^, which is characterized by predominant protein-related absorption bands. This region is populated by a series of indicative absorption bands that are highly specific for the molecular vibrations of proteins (although they overlap fewer intensive signals of other BP/BB constituents exhibiting signals in this region, such as those of sugars, as elaborated by Prđun et al. [16]).

A medium-intensity absorption band observed at 1645 cm^−1^ is typical for protein structures (β-sheet) and can be assigned to the Amide I band, which primarily comprises stretching vibrations of the C=O (70–85%) and C–N groups (10–20%) [51,52]. As elaborated in detail by Prđun et al. [16], this band position is also characteristic for the molecular vibrations of water (H–O–H deformation vibration) and lipids (COO– and C=C stretching vibration). Expectedly, the overlapping effects of the aforementioned vibrations were reflected in this region. In order to elucidate these overlaps and exclude the water signal, an additional spectral analysis was performed on lyophilized samples, together with the water loss evaluation, as elaborated in detail below.

Other protein-related signals attributed to the protein side chain COO– stretching vibrations can be observed at 1413, 1370, and 1341 cm^−1^, while the absorption band arising at 1238 cm^−1^ occurs due to the Amide III band, which comprises 30% of N–H bending, 30% of C–N stretching, 10% of C–O stretching, and 10% of O=C–N bending vibrations [51]. The high similarity of the BP and BB spectral pattern, with regard to the aforementioned protein-related signals occurring in the spectral region from 1700 to 1190 cm^−1^ (with slightly higher intensities of corresponding analyte signals in BB), indicates a compositional similarity/stability in terms of the proportion of proteins in the analyzed BP and BB samples and confirms the successful conservation of bee pollen by transformation into bee bread. An exception in terms of the observed differences in this spectral envelope is represented by a higher absorbance intensity of two neighboring signals at 1544 cm^−1^ and 1515 cm^−1^ associated with C=C stretching vibrations of the aromatic structures of lipids in the BP sample. As reported by Conte et al. [53], in addition to the predominant triglycerides, diglycerides, and fatty acids, the lipid fraction of BP also contains a high content of phospholipids, tocopherols, and phytosterols. These compounds represent more complex constituents of the BP lipid fraction and are characterized by aromatic ring structures, which is reflected in the spectrum in terms of the mentioned signals. Along with the aforementioned absorptions at 2925 and 2854 cm^−1^, specific for aliphatic chains of lipids that proved to be higher in BP, the intensity of the adjacent bands arising at 1544 and 1515 cm^−1^ also reflects a higher proportion of lipids in BP compared to that found in BB. This is in accordance with data from the available scientific literature, according to which the proportion of lipids in BP ranges from 0.41 to 24% [1,14,18], and in BB from 1.9 to 11.6% [1,3]. The higher content of lipids in BP compared to that found in BB can be explained by a particular mechanism of action of microorganisms during the BP-to-BB transformation process. Generally, several groups of microorganisms are involved in the conversion of BP to BB, mainly *Pseudomonas*, LAB, and microscopic fungi. They are all known to produce lipases and hydrolyze lipids [56,57]. Although LAB are considered to be weakly lipolytic compared to *Pseudomonas* and microscopic fungi, their lipolytic activity is also well-documented [58]. Therefore, all three groups of microorganisms may play an important role in the lipid degradation of BP observed in our study. 

It should be noted here that the Amide II band, which comprises N–H bending and C–N stretching vibrations, and typically absorbs between 1510 and 1580 cm^−1^, is overlapped by the former lipid-based C=C stretching that arises at 1544 and 1515 cm^−1^, as elaborated by Prđun et al. [16]. These C=C stretching vibrations (both or individual) are often (mis)assigned primarily as the Amide II band of proteins; however, it has to be emphasized that the position, shape, and intensity of the adjacent bands at 1544 and 1515 cm^−1^ observed in the BP/BB spectrum do not correspond to the Amide II band characteristics. Indeed, Amide II typically absorbs between 1510 and 1580 cm^−1^ (mostly around 1550–1560 cm^−1^) [51], but it appears as a single medium-broad and conspicuous band, not in the form or as a part of narrow adjacent bands (such as those observed in the BP/BB spectra). 

Along with the above-mentioned spectral features reflecting differences in the water content, the most prominent differences between the BP and BB spectra were observed in the fingerprint region from 1180 to 750 cm^−1^ (Figure 8).

As accentuated in Figure 8, the FTIR-ATR spectra of BP and BB differ in terms of both alterations in the position and the intensity of the absorption bands. Namely, it was observed that the intensity of the absorption bands at 994 cm^−1^ and 922 cm^−1^, the sucrose-characteristic signals assigned to C–O–C stretching vibrations and C–C stretching vibrations, respectively [50,59,60], are significantly lower in BB compared to that observed in BP. This indicates a breakage of both C–O–C and C–C glycosidic bonds of sucrose during the fermentation of BP (decomposition of sucrose), which causes conformational changes in the sugar fraction in BB, as further explained and presented in Figure 8. Moreover, a significant decrease in the sucrose signal at 994 cm^−1^ (to the level of being barely noticeable) in BB confirms the predominance of monosaccharides in BB compared to the predominant sucrose in BP, as it is known that there is little absorption at this position (near 1000 cm^−1^) in the monosaccharides [49].

As elaborated by Bakour et al. [6], sucrose appears in a low proportion in bee bread due to the fact that, during fermentation, sucrose is used by bacteria and yeasts as an energy source. However, in order to utilize sucrose, LAB and yeasts must first convert it to fermentable monosaccharides (glucose and fructose) by invertases (sucrase and saccharase). The mentioned spectral observations reflecting low sucrose content in BB are further supported by data from the available scientific literature, according to which the proportion of sucrose in BP ranges from 5.87 to 22.04% [16], while in BB it is found almost in trace amounts [6], i.e., 0.845−1.49% [1].

Furthermore, a comparative spectral analysis of BP vs. BB has revealed that considerable amounts of glucose are present in both BP and BB, but a slightly higher proportion was found in BP, which is reflected in the spectra of the analyzed samples. Namely, the absorption band at 922 cm^−1^, characteristic for C–C stretching vibrations of glucose, is more intense in the spectrum of bee pollen, while these differences are less pronounced at the position of the second band, characteristic for glucose, at 1031 cm^−1^. The aforementioned observations can also be associated with previously published data on the proportion of glucose, which in bee pollen averages at 12.4% [16] and at 5.7% in bee bread [6].

Noticeable spectral differences between BP and BB were also observed in terms of higher absorbance intensities of bands observed in BB at 865, 817, and 776 cm^−1^. These absorption bands are attributed to the molecular vibrations of fructose. The first signal appears due to the C–C stretching vibrations, while the other two correspond to the C–C–H deformation vibrations [49,61]. A higher proportion of fructose in BB compared to BP is supported by the literature data emphasizing fructose as the most abundant sugar in BB (up to 19.73%), followed by 8.82−12.40 of glucose, and 0.845−1.49% of sucrose [1]. 

The overall spectral variations in this sugar-based spectral envelope (1200–750 cm^−1^) indicate a lower content of sucrose (and to a lesser extent glucose) and a higher proportion of fructose in BB compared to that found in BP, which is primarily a consequence of the hydrolysis of sucrose to glucose and fructose by invertases produced by honey bees and various groups of microorganisms. Glucose and fructose are, thus, the main fermentable sugars in bee pollen, and, usually, glucose is fermented much faster than fructose, which is mainly related to the preferential use of glucose by microorganisms [62]. 

Polysaccharides and oligosaccharides are considered minor BP constituents, and, as such, they contribute little to the overall spectral features associated with the BP carbohydrate fraction. The oligosaccharides in BP (such as isomaltose, raffinose, trehalose, and erlose) are represented in a low proportion (<1.5%, with an exception of maltose, which ranges from 0.16 to 6.03%), while cellulose (as the main component of the layers of pollen grains) is the most abundant BP polysaccharide, comprising on average 3–4% of BP [1,14].

**Figure 9:**
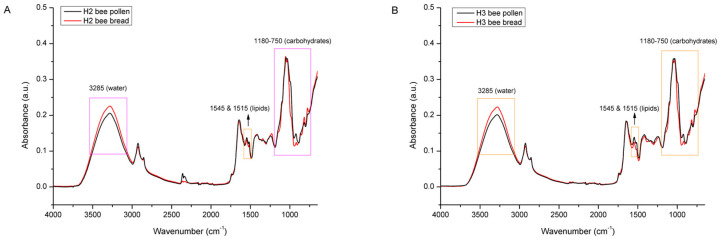
Average FTIR-ATR spectrum of bee pollen vs. bee bread from experimental colony H2 (**A**) and H3 (**B**), representing comparative spectral features with the assignation of major underlying molecular vibrations in the whole spectral region (4000–600 cm^−1^).

The same trend of chemical changes (differences in the proportion of water and the changed profile of the sugar fraction, and to a lesser extent the lipid fraction) were also observed with comparative spectral analysis of BP vs. BB from experimental colonies H2 (Figure 9A) and H3 (Figure 9B).

**Figure 10:**
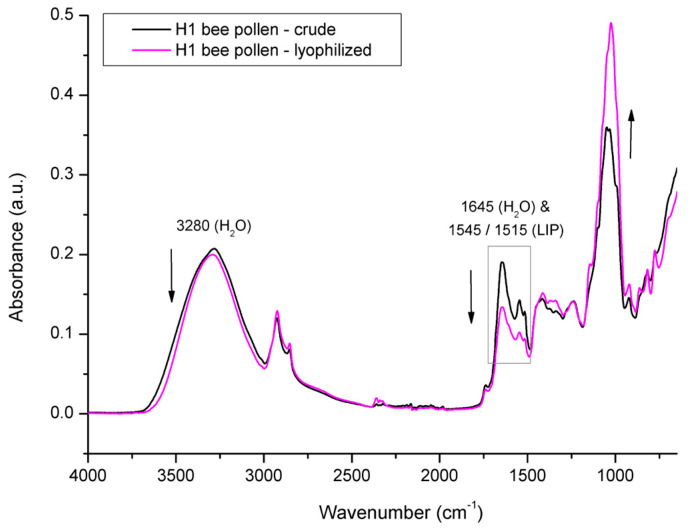
Average FTIR-ATR spectrum of crude bee pollen vs. lyophilized bee pollen from experimental colony H1, representing comparative spectral features.

In order to elucidate the spectral effects related to overlapping water signals, the investigated BP and BB samples were lyophilized and reanalyzed with FTIR-ATR spectroscopy. Figure 10 shows the comparative spectral features of crude vs. lyophilized BP from colony H1. As freeze-drying (lyophilization) causes dehydration of the treated samples, it has affected the water signals in the BP spectrum, thus enabling the confirmation of the water contribution to particular absorption bands. The major spectral effect observed in the lyophilized BP compared to the crude BP was reflected in the decrease in water signals at 1645 cm^−1^ and 3280 cm^−1^ and a simultaneous increase in the intensity of the absorption bands related to the sugar fraction in the spectral region between 1200 and 700 cm^−1^ (primarily due to the changed concentration of monosaccharaides in BP, i.e., glucose and fructose). The higher concentration of monosaccharides in the lyophilized BP sample is also reflected in the spectral region between 1500 and 1200 cm^−1^, in terms of a minor intensity increase in signals observed at 1410 cm^−1^ and 1342 cm^−1^, corresponding to the C–O–H deformation of glucose and fructose, respectively [50,61].

**Figure 11:**
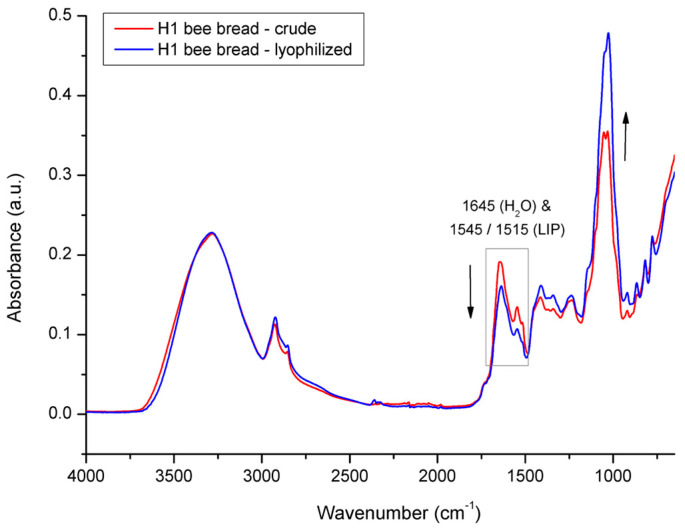
Average FTIR-ATR spectrum of crude bee bread vs. lyophilized bee bread from experimental colony H1, representing comparative spectral features.

The same mode of freeze-drying action was carried out on lyophilized vs. crude BB (Figure 11); however, the decrease in intensity of both water signals in BB was less pronounced, which indicates less intensive water loss in BB compared to that of BP during the same lyophilization treatment. These spectral observations were in compliance with the results on water loss obtained gravimetrically for the BP and BB samples. Namely, the water loss determined for BP from experimental colony H1 was 14.6% and was 10.5% for BB. These results indicate a higher proportion of residual water in the lyophilized BB compared to that of the lyophilized BP; however, this still needs to be elucidated, but it can be assumed this is a consequence of differently hydrated forms of constituents in BP and BB. It was also observed that the water loss in the BP and BB samples was followed by a decrease in the signal intensity at 1645 cm^−1^, which was more pronounced compared to the decrease observed at 3280 cm^−1^. This can be explained by the contribution of O–H stretching vibrations of both water and carbohydrates to the absorption band at 3280 cm^−1^, which somewhat neutralizes the intensity decrease in this band. Similar spectral effects in relation to water- vs. sugar-associated vibrations have been observed by other authors [49,50,61].

The spectral analysis of crude vs. lyophilized BP has also revealed changes in the lipid fraction. Moreover, it was observed that lyophilization also causes the degradation of a particular group of lipids, i.e., lipids with aromatic structures represented by absorption bands occurring at 1544 cm^−1^ and 1515 cm^−1^ whose intensities were found to be decreased in both BP and BB. Similar effects of lyophilization and/or other drying methods on the lipid fraction of BP were observed by other authors [53,63,64,65]. For example, Conte et al. [53] reported that tocopherol shows a significant decrease after freeze-drying treatment, i.e., α -tocopherol, δ-tocopherol, and γ-tocopherol showed a reduction of 60%, 75%, and 90%, respectively. 

The degradation of lipids caused by lyophilization was also reported for other types of organic specimens, such as plants (e.g., tarragon), lipomers and various liposomal formulations, algae, and vegetable oils [66,67,68,69,70]. Along with the water decrease, the weaker intensity of signal at 1645 cm^−1^ in the lyophilized BP/BB samples is probably partially related to a lipid decrease as well, given that the COO– and C=C stretching vibration of lipids are overlapped by H–O–H deformation vibration of water in this spectral region. It can be assumed that lyophilization also affects other less represented lipids that are a part of pollen grains from which BP is comprised, such as glycophospholipids, sphingolipids, sterols, and oxylipids, as well as lipopolysaccharides [71]; however, these minor BP constituents should be further investigated, as they are rarely mentioned in the literature covering BP/BB.

**Figure 12:**
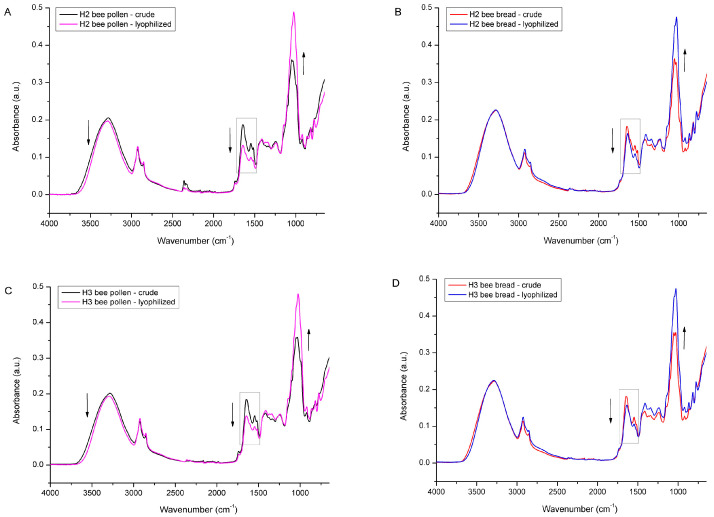
Average FTIR-ATR spectrum of crude bee pollen vs. lyophilized bee pollen (**A**) and crude bee bread vs. lyophilized bee bread (**B**) from experimental colony H2; crude bee pollen vs. lyophilized bee pollen (**C**) and crude bee bread vs. lyophilized bee bread (**D**) from experimental colony H3.

All of the above-described spectral effects (a decrease in water and lipid content, as well as an increase in sugar fraction due to changes in the concentration of individual constituents) were also observed in the lyophilized BP and BB samples from experimental colonies H2 and H3, as presented in Figure 12. Also, the determined water loss for those samples was similar to the results obtained for the samples from H1, i.e., the water loss determined in BP from colony H2 was 14.67% and 9.38% for BB, while the BP from the H3 colony exhibited 14.78% water loss and 10.89% in BB.

The overall results of the spectral analysis conducted on the BP/BB samples collected from three honey bee colonies confirm the same mode of chemical alterations during the transformation process of bee pollen into bee bread. These changes are primarily the result of lactic acid fermentation, but it can be assumed that the presence of particular enzymes in bee bread also contributes to sugar-related modifications. Also, the presented results confirm the effectiveness of the bee pollen preservation process, given that the basic chemical composition of BP remains preserved but enriched in the BB form by numerous beneficial elements (the presence of lactic acid, a lower pH, better nutrient bioavailability, and protective/antimicrobial properties).

## 4. Conclusions

In this study, we have focused our research on the investigation of the in-hive environment (honeycomb) and chemical changes that occur during the transformation of bee pollen into bee bread by monitoring the metric and spectral aspects of this process on three experimental colonies of Carniolan honey bee (*A. m. carnica*). The results have shown that the average depth of the honeycomb (worker) cells was 11.00 mm, and that the bees stored bee pollen up to an average of 7.85 mm of cell depth, thus covering between 23 and 34 (71.4%) of the honeycomb cell—to be more specific and mathematically accurate, 1724 of the honeycomb cell. This finding represents the first report, i.e., the first metric data, on the cell depth at which *A.m. carnica* honey bees store their bee bread. The results of the complementary cell measurements have revealed that the smallest side length of the analyzed honeycomb cells ranged from 9.12 to 10.63 mm, with an average of 9.74 mm, while the angle of the hexagonal cell ranged from 117.8° to 119.2°, with an average of 118.4°. These metric data may be employed to investigate the hive-level distribution and storage of bee bread and to estimate the annual production and economic value of bee bread production in general.

We have also provided a detailed assignation of the molecular vibrations observed in bee pollen and bee bread (crude and lyophilized) and elucidated the additional aspects of bee-pollen-to-bee-bread conversion based on a comparative spectral overview of both substances collected simultaneously from three experimental *A. m. carnica* colonies. The FTIR-ATR analysis has revealed a unique spectral pattern of both the bee pollen and the bee bread samples, indicating compositional changes primarily reflected in a higher water content and an altered composition of the sugar fraction (a lower content of sucrose and a higher proportion of fructose in the bee bread compared to the bee pollen), and, to a lesser extent, the lipid fraction (a lower lipid content in the bee bread compared to the bee pollen). Further investigation should be focused on additional microbiological assaying aiming to determine the detailed background mechanisms of the bee-pollen-to-bee-bread transformation pathway.

## Figures and Tables

**Table 1:** Descriptive statistics for the depth of *A. m. carnica* honeycomb cells (*n* = 30 cells/colony).

Depth of Honeycomb Cell (mm)	Experimental Colony			
	H1	H2	H3	Total
Average	10.99	11.01	11.01	11.00
Minimum	9.71	8.97	9.37	9.35
Maximum	12.05	11.98	12.36	12.13
Standard deviation	0.57	0.81	0.79	0.72

**Table 2:** Descriptive statistics for the depth of honeycomb cells in which honey bees store bee bread (*n* = 100 cells/colony).

Depth of the Cell in Which Bees Store Bee Bread (mm)	Experimental Colony			
	H1	H2	H3	Total
Average	7.38	8.14	8.02	7.85
Minimum	5.19	5.25	5.20	5.21
Maximum	10.26	10.71	9.72	10.23
Standard deviation	1.19	1.09	1.00	1.09

**Table 3:** Descriptive statistics for the smallest side length of honeycomb cells in which honey bees store bee bread (*n* = 30 cells/colony).

Side Length of the Honeycomb Cell (mm)	Experimental Colony			
	H1	H2	H3	Total
Average	9.73	9.63	9.87	9.74
Minimum	9.12	9.12	9.11	9.12
Maximum	10.47	10.66	10.76	10.63
Standard deviation	0.39	0.35	0.48	0.41

**Table 4:** Descriptive statistics for the angle of hexagonal honeycomb cells in which honey bees store bee bread (*n* = 30 cells/colony).

Angle (∡) of Hexagonal Honeycomb Cell (°)	Experimental Colony			
	H1	H2	H3	Total
Average	118.5	118.4	118.4	118.4
Minimum	117.8	117.8	117.7	117.8
Maximum	119.1	119.2	119.4	119.2
Standard deviation	0.37	0.39	0.41	0.39

## Data Availability

Data are contained within the article.
